# When is There no Benefit in Performing a Biopsy in the Suspicion of Intraepithelial Lesions of the Cervix?

**DOI:** 10.1055/s-0042-1744212

**Published:** 2022-05-16

**Authors:** Marília Porto Bonow, Luiz Martins Collaço, Ana Paula Percicote, Rita Maira Zanine

**Affiliations:** 1 Department of Obstetrics and Gynecology, Clinics Hospital of the University of Paraná, Curitiba, PR, Brazil; 2 Department of Pathology of Clinics Hospital of the University of Paraná, Curitiba, PR, Brazil; 3 Department of Gynecology and Obstetrics, Lower Genital Tract Disease and Colposcopy Sector, Clinics Hospital of the University of Paraná, Curitiba, PR, Brazil

**Keywords:** cervical intraepithelial neoplasia, colposcopy-directed biopsy, conization, cervical cancer, neoplasia intraepitelial cervical, biópsia colpodirigida, conização, câncer cervical

## Abstract

**Objective** To evaluate whether colposcopy-directed biopsy is necessary to increase the accuracy of diagnosing cervical intraepithelial lesions in relation to colposcopy.

**Methods** We performed a retrospective, observational study by analyzing medical records obtained from Hospital de Clínicas do Paraná from February 2008 to February 2018. Patients with results of Pap tests, colposcopy, colposcopy-directed biopsy, and surgical procedures (high-frequency surgery or cold conization) were included. Data such as quadrants involved during colposcopy and age differences were also analyzed.

**Results** A total of 299 women were included. Colposcopy was found to have an accuracy rate of 76.25% (95% confidence interval [CI], 71.4–81.1). Among the highest-grade lesions, the accuracy rate was 80.5% (95% CI, 75.7–85.3). The accuracy rates for biopsy were 79.6% (95% CI, 75–84.2) and 84.6% (95% CI, 80–89.1) for the highest-grade lesions. High-grade lesions were accurately confirmed in 76.9% and 85% of patients with 1 and 2 or more affected quadrants, respectively. For women younger than 40 years, the accuracy rates were 77.6% and 80.8% for colposcopy and biopsy, respectively. For women 40 years or older, the accuracy rates were 72.5% and 76.3% for colposcopy and biopsy, respectively.

**Conclusion** There is no difference between the accuracy of colposcopy and that of biopsy in diagnosing cervical intraepithelial lesions in relation with the result of conization. The patients who received the greatest benefit when biopsy was not performed were those with high-grade lesions at colposcopy, a lesion involving 2 or more quadrants, and those younger than 40 years.

## Resumo

**Objetivo** Avaliar se a biópsia colpodirigida é necessária para aumentar a acurácia diagnóstica nas lesões intraepiteliais de colo uterino em relaçãoà colposcopia.

**Métodos** Estudo retrospectivo, observacional, incluindo pacientes submetidas a colposcopia, biópsia colpodirigida, e procedimento cirúrgico (cirurgia de alta frequência ou conização a frio), no período de fevereiro de 2008 a fevereiro de 2018, no Hospital de Clínicas da Universidade Federal do Paraná. Dados como número de quadrantes da lesão presentes na colposcopia, número de fragmentos retirados nas biópsias e diferenças por idade também foram analisados.

**Resultados** Um total de 299 mulheres foram incluídas. Foi encontrada uma acurácia de 76,25% (intervalo de confiança [IC] 95% 71,4–81,1) entre a colposcopia e a conização, sendo 80,5% % (IC 95% 75.7–85.3) nas lesões de maior grau. A acurácia da biópsia foi de 79,6% (IC 95% 75–84,2), sendo 84,6% (IC 95% 80–89,1) nas lesões de maior grau. Pacientes com 1 quadrante acometido tiveram confirmação de 76,9% nas lesões de maior grau, enquanto as com 2 quadrantes acometidos apresentaram o mesmo resultado em 85% dos casos. A acurácia com a biópsia de 1 fragmento foi de 78% e com 2 ou mais fragmentos 80%. Para mulheres com menos de 40 anos, a acurácia foi de 77,6% e 80,8% para colposcopia e biópsia, respectivamente. Para mulheres com 40 anos ou mais, a acurácia foi de 72,5% e 76,3% para colposcopia e biópsia, respectivamente.

**Conclusão** Não há diferença entre a acurácia da colposcopia e a da biópsia colpodirigida no diagnóstico de lesões intraepiteliais cervicais em relação ao resultado da conização. As pacientes que tiveram o maior benefício quando a biópsia não foi realizada foram as que apresentaram lesão de alto grau na colposcopia e aquelas com menos de 40 anos, não existindo benefício em realizar biópsia previamente a conização neste grupo de pacientes.

## Introduction

Adequate management of women with cervical intraepithelial neoplasia is a critical component in the prevention of cervical cancer. 1 A major challenge involved in the treatment of precursor lesions and cervical cancer is deciding which methods to use to make the final diagnosis. Identification of the location and extent of the lesion, biopsies of areas with the highest suspicion of malignancy, and subsequent performance of the most appropriate surgical procedure can be decisive factors leading to the best prognosis. However, the steps required to identify these lesions have been increasingly questioned. Therefore, researchers must determine which patients would benefit most from particular strategies.

Pap tests are not sufficiently specific to justify treatment for women with abnormal results. 2 3 4 Colposcopy has greater sensitivity and is best for differentiating between low-grade and high-grade diseases of the normal cervix; its contributions are undeniable, but its diagnostic accuracy has since been questioned due to issues associated with random biopsies and biopsy numbers. 5 6 Therefore, histology should remain the gold standard for treating suspicious lesions. 5 7 8 However, when the histopathological results of biopsies and conizations are analyzed, differences between results can occur, leading to questions about factors related to diagnostic agreement. 9 10 Agreement between colposcopy and conization results, mainly for high-grade lesions, and differences between histopathological results create doubts about the benefits of biopsy. Therefore, different strategies have been studied to determine effective and lower-cost treatments, and some authors believe that the combination of different findings can lead to a correct diagnosis. 11 The “see and treat” protocol is a strategy for patients with Pap test and colposcopy results indicating suspected high-grade lesions; these patients undergo a loop electrosurgical excision procedure during a single clinical visit. This strategy was accepted internationally due to the lower costs for the health system, especially in places with limited resources; decreased anxiety for patients; and greater patient compliance. 12 In Brazil, variations of this strategy are necessary because of the use of the Unified Health System (SUS, in the Portuguese acronym), which is a public health system in which bureaucracy does not allow procedures to be performed without an audit, release of the procedure, and prior scheduling. A two-stage treatment strategy involves conization that is scheduled within a short period of time after colposcopy results indicating high suspicion of malignancy.

## Methods

We evaluated whether biopsy is necessary to increase the accuracy of diagnosing intraepithelial lesions of the uterine cervix by comparing the accuracy of colposcopy and colposcopy-directed biopsy. We assessed whether the number of lesion-involved quadrants is related to greater diagnostic accuracy, and whether there is a difference between colposcopy and biopsy accuracy for patients stratified by age (younger than 40 years or 40 years or older). We also aimed to define which patients can benefit from the elimination of biopsy.

A retrospective, observational study by analyzing medical records was performed. Participants were recruited at the Pathology Service of the Lower Genital Tract of the Hospital de Clínicas da Universidade Federal do Paraná in Curitiba, Brazil. Patients examined from February 2008 to February 2018 were included.

Only women attended at the SUS who had been referred to the service due to abnormal Pap test results or with cervical lesions identified during the examination at the time of data collection were included. All patients underwent colposcopy, biopsy, and conization (high-frequency surgery or cold conization) at our service.

Patients with negative Pap test results, low-grade squamous intraepithelial lesions (LSIL), or atypical squamous cells of undetermined significance (ASCUS) underwent biopsy when they had a high-grade colposcopic impression or persistent low-grade colposcopic impression. Patients who had negative biopsy but persistent high- or low-grade colposcopy results also underwent conization. Only cases with available histopathological material and evaluable for review were included.

The colposcopy results were classified according to the 2011 International Federation for Cervical Pathology and Colposcopy. 13 Major and minor findings during the colposcopy examination were classified according to the highest-degree findings. All procedures were performed by resident and specialist physicians who were always supervised by the same physician specialized in pathology of the lower genital tract with 40 years of experience.

Regarding histopathological analyses, the official report released by the service was initially considered. To increase the reliability of our study, all slides obtained after biopsies and surgical procedures were reviewed by a single experienced pathologist (pathologist 1); in the case of divergence between the original report and the report of pathologist 1, the slides were analyzed again by another experienced pathologist (pathologist 2). Pathologists performed the analysis without knowledge of the original reports; they only knew that the material was from a biopsy or conization. In case of divergence between the three results, the highest-degree finding was considered the result. All results were based on the final review of the procedures and a comparison of the results among pathologists.

The gold standard was considered histopathological diagnosis using conization, always considering important factors, as in the case of lesions that may have been removed completely in the biopsy. The results were classified as normal, characteristic low-grade lesions, characteristic high-grade lesions or carcinoma in situ, microinvasive carcinoma, or invasive carcinoma. The time (months) between biopsy and conization was reported.

For the statistical analysis, minor and miscellaneous findings of colposcopy were considered consistent with low-grade biopsy results. Major findings or suspected invasion suggested by colposcopy were considered consistent with high-grade results, microinvasive carcinoma, or invasive carcinoma found by biopsy. The same analysis was performed using conization results. Patients with major findings according to colposcopy or high-grade findings according to biopsy were considered overtreated when they had negative or low-grade results according to conization, noting an important point that the biopsy may have removed HSIL lesion. Patients with minor lesions according to colposcopy or low-grade findings according to biopsy were considered overtreated when they had negative conization results (this group of patients was submitted to conization when they had persistent Pap test changes or a persistent low-grade colposcopic impression).

The severity of the findings was assessed, as were data such as the number of quadrants involved in colposcopy (from 1–4), which were analyzed using drawings in the handbooks; when the pattern was not identified, it was classified as not informed.

The study results are described as means, standard deviations, minimum values, and maximum values (quantitative variables), or as frequencies and percentages (categorical variables). The Fisher exact test results and estimated odds ratios (ORs) were used to assess the association between two dichotomous categorical variables. To analyze the quality of the colposcopy and biopsy in terms of predicting the results of conization (gold standard), sensitivity, specificity, and accuracy values were estimated, and 95% confidence intervals (CI) were presented; *p* < 0.05 indicated statistical significance. Data were organized in an Excel (Microsoft Corp., Redmond, WA, USA) spreadsheet and analyzed using the Stata/SE computer software, version 14.1. (StataCorp LP, College Station, TX, USA). The study protocol was approved by the Internal Review Board of Universidade Federal do Paraná (Curitiba, PR, Brazil; CAAE: 46807015.1.0000.0096).

## Results

During the study period, a total of 299 patients were analyzed. The mean age was 33.9 years, and 219 subjects (73.2%) were younger than 40 years. Ten (3.3%) women were postmenopausal. Thirty-three (11.1%) had a history of a sexually transmitted infection, 199 (67.7%) had 1 to 3 pregnancies, and 218 (74.1%) had at least 1 vaginal delivery. The preceding Pap test results were as follows: 190 (63.5%) cases of high-grade squamous intraepithelial lesions (HSIL), atypical squamous cells cannot exclude high-grade squamous intraepithelial lesions (ASC-H), or atypical glandular cells (AGC); 55 (18.4%) cases of LSIL or normal results; and 54 (18.1%) cases of ASCUS. All cervical smears were performed using the conventional Pap test. Colposcopy diagnosed the following: 238 (79.6%) abnormal findings (high-grade); 25 (13.0%) normal findings; 35 (11.7%) abnormal findings (low-grade); 24 (8%) findings indicating suspected invasion; and 2 (0.7%) miscellaneous findings. The histological results of colposcopy-directed biopsy were as follows: 245 (81.9%) cases of high-grade lesions or adenocarcinoma in situ (AIS); 53 (17.7%) cases of no lesions or low-grade lesions; and 1 (0.3%) case of microinvasive cervical cancer. The histological results of conization were as follows: 214 (71.6%) cases of high-grade or AIS; 68 (22.7%) cases of no lesions or low-grade lesions; and 17 (5.7%) cases of microinvasive or invasive cervical cancer. Table 1 shows the results of conization stratified by the colposcopic impressions and biopsy results. Table 2 shows the prediction of conization based on colposcopic impressions and biopsy results. The accuracy rates of colposcopy and biopsy were similar. Colposcopy had an accuracy of 76.25% (95% CI, 71.4–81.1), and biopsy had an accuracy of 79.6% (95% CI, 75–84.2) ( *p* = 0.275). Among the highest-grade lesions, 80.5% (95% CI, 75.7–85.3) were found by colposcopy and 84.6% (95% CI, 80–89.1) were found by biopsy (positive predictive value). The sensitivity of colposcopy was 91.3% (95% CI 87.7–95), and that of biopsy was 90% (95% CI, 86.2–93.9%; *p* = 0.742). Specificity was higher when biopsy was performed (biopsy: 44.1%; colposcopy: 25%; *p* = 0.031).

**Table 1:** Results of colposcopies, biopsies, conizations

Conization			
Colposcopic impression	High-grade/microinvasive or invasive cervical cancer	Low-grade/negative	Total
High-grade/suspected invasion	211	51	262
No lesion/low-grade/miscellaneous	20	17	37
Total	231	68	299
**Biopsy**			
High-grade/Microinvasive or invasive cervical cancer	208	38	246
No lesion/Low-grade	23	30	53
Total	231	68	299

**Table 2:** Colposcopy and biopsy sensitivity, specificity, and accuracy

Variables	Colposcopy (95% CI)	Biopsy (95% CI)
Sensitivity	91.3% (87.7–95) NS	90.0% (86.2–93.9) NS
Specificity	25.0% (14.7–35.3)*	44.1% (32.3–55.9)*
Accuracy	76.3% (71.4–81.1) NS	79.6% (75–84.2) NS

*McNemar test, *p* < 0.05

The probability rate of false-negative test results was 8.7% (95% CI, 5–12.3) for colposcopy, and it was 10% (95% CI, 6.1–13.8) for biopsy. The rate of overtreatment for high-grade colposcopy findings was 19.5% (95% CI, 14.7–24.3) for colposcopy, and it was 15.4% (95% CI, 10.9–20) for biopsy. The rate of general overtreatment was ∼ 18% for both diagnostic methods. There was no statistical difference between the results. The average time between biopsy and conization for cases with concordant results was 5.9 months. For divergent cases, the time between biopsy and conization was longer when conization presented a greater degree of malignancy than biopsy (mean, 7.3 months; *p* = 1). Data regarding the number of quadrants involved using colposcopy were available for 291 cases; 17 of these cases involved only 1 quadrant and 274 cases involved 2 or more ( Table 3 ). For patients with involvement in 1 quadrant, accuracy was 70.6%, sensitivity was 83.3%, and specificity was 40%. For patients with involvement in 2 or more quadrants, accuracy was 80.3%, sensitivity was 90.6%, and specificity was 45.2%. Regarding the highest-grade findings discovered by biopsy, conization confirmed 76.9% with involvement in one quadrant and 85% with involvement in 2 or more quadrants. The probability rate of false-negative results was 16.7% with involvement in 1 quadrant, and it was 9.4% with involvement in 2 or more quadrants. A statistical comparison of such data was not possible due to the small number of patients with involvement in one quadrant.

**Table 3:** Number of quadrants, colposcopies, and conizations

Conization			
1 quadrant	High-grade/microinvasive or invasive cervical cancer	Low-grade/negative	Total
High-grade/suspected invasion	10	3	13
Low-grade/miscellaneous	2	2	4
Total	12	5	17
**2 or more quadrants**			
High-grade/suspected invasion	192	34	226
Low-grade/miscellaneous	20	28	48
Total	212	62	274

Conization diagnosed 17 cases of microinvasive or invasive carcinoma (5.7%). Most patients had previous HSIL, ASC-H, or AGC diagnosed by Pap tests (76.5%). Pap tests diagnosed ASCUS in 17.6%. Sixteen cases (94.1%) of high-grade findings or suspected invasion were diagnosed with colposcopy; of these, 14 (87.5%) involved two or more quadrants. Biopsy found 15 (88.2%) high-grade findings and 2 negative results. The only case of a low-grade lesion diagnosed with colposcopy also had negative biopsy results. Table 4 demonstrates the results of colposcopy, biopsy, and conization stratified by age. Of 192 high-grade lesions found by colposcopy in women younger than 40 years, 158 (82.3%) received confirmation by conization (95% CI, 76.9–87.7). There were 183 cases of lesions diagnosed by biopsy, with 157 (85.8%) cases confirmed by conization (95% CI, 80.7–90.9). Overtreatment occurred in 17.7% of these cases (95% CI, 12.3–23.1) according to colposcopy, and in 14.2% (95% CI, 9.1–19.3) according to biopsy. Among younger patients, colposcopy had accuracy of 77.6% (95% CI, 86.4–95.1), sensitivity of 91.3% (95% CI, 87.1–95.5), and specificity of 26.1% (95% CI, 13.4–38.8). In these same patients, biopsy had accuracy of 80.8% (95% CI, 75.6–86), sensitivity of 90.8% (95% CI, 86.4–95.1), and specificity of 43.5% (95% CI, 29.2–57.8).

**Table 4:** Colposcopy and biopsy results leading to conization stratified by age

Conization			
Colposcopic impression	High-grade/microinvasive or invasive cervical cancer	Low-grade/negative	Total
< 40 years			
High-grade/suspected invasion	158	34	192
No lesion/low-grade/miscellaneous	15	12	27
Total	173	46	219
≥ 40 years			
High-grade/suspected invasion	53	17	70
No lesion/low-grade/miscellaneous	5	5	10
Total	58	22	80
**Biopsy**			
<40 years			
High-grade/microinvasive or invasive cervical cancer	157	26	183
No lesion/low-grade	16	20	36
Total	173	46	219
≥ 40 years			
High-grade/microinvasive or invasive cervical cancer	51	12	63
No lesion/low-grade	7	10	17
Total	58	22	80

Fifty-three (75.7%) of 70 cases of high-grade lesions diagnosed by colposcopy were confirmed in women older than 40 years. Biopsy diagnosed 63 cases of high-grade lesions, and 51 (81%) were confirmed. Overtreatment rates of these cases were 24.3% for colposcopy and 19% for biopsy. Colposcopy accuracy among older patients was 72.5%, with sensitivity of 91.4% and specificity of 22.7%. Biopsy had accuracy of 76.3%, sensitivity of 87.9%, and specificity of 45.5%. A statistical analysis comparing younger patients with the older ones was not performed because of the small number of patients older than 40 years.

## Discussion

The accuracy of diagnostic tests to identify cervical precursor lesions is important for reducing mortality. Our results show no significant difference between the accuracy of colposcopy and that of colposcopy-directed biopsy, and there are cases that do not require biopsy before conization.

The “see and treat” strategy often cannot be performed in Brazil due to administrative issues. A two-stage treatment strategy can be beneficial because fewer visits are required for definite diagnoses; the patient waits for histologic results of the biopsy and schedules an additional appointment for definite treatment if indicated. This is time-consuming and costly. Reducing hospital visits generates savings in commuting and services (approximately $1,000 for every 100 biopsies avoided) and creates new appointment vacancies for other patients.

We found increased diagnostic accuracy for higher-grade lesions, and there was no significant difference between colposcopy and biopsy compared with conization. Patients with high-grade lesions according to colposcopy results have a greater chance of having these results confirmed by both biopsy and conization, demonstrating that biopsy can be dismissed to diagnose high-grade lesions. A 2010 study involving colposcopy-guided biopsies immediately before the surgical procedure found no diagnostic benefit for patients with high-grade lesions. 13 14 Booth et al. 15 found no significant difference between colposcopy and biopsy, with 80.6% agreement for histopathological results.

According to the literature, identification of an acetowhite lesion is a highly sensitive indicator of high-grade lesions or cancer. However, the specificity of this finding seems low, and directed biopsy is required to guide subsequent therapy. 2 In our study, there was no significant difference between the sensitivity of biopsy and that of colposcopy ( *p* = 0.742), indicating that the tests are similar in their abilities to find lesions, such as during screening tests, and guide conization. Mitchell et al. 16 performed a large meta-analysis and found that colposcopy had sensitivity ranging from 87 to 99% and specificity ranging from 23 to 87%.

Specificity was low for biopsy and colposcopy, but it was higher for biopsy (44.1%) than for colposcopy (25%) ( *p* = 0.031). Biopsy is more capable of confirming the absence of lesions or low-grade lesions; therefore, it should be performed when high-grade lesions are suspected in such patients.

Colposcopy-directed biopsy had slightly higher rates of false-negative results than colposcopy, but the difference was not statistically significant (10% versus 8.7%). This demonstrates that even when two histopathological examinations are performed, there may be differences in their findings, and failure to detect more advanced lesions may occur. This is in accordance with the literature, indicating that biopsy can confirm high-grade lesions but cannot exclude them. 14

Overtreatment can lead to unnecessary treatments and increased costs. The general overtreatment rates were similar for the two groups studied (19%) and similar to that found in the literature (23%) when “see and treat” was performed for patients with different degrees of Pap test results. 17 Although there was no statistical difference, overtreatment occurred more often with colposcopy than with biopsy for higher-grade lesions (19.4% vs 15.4%) because the lesions were probably removed during biopsies before conization. Because the initial biopsy may have removed the entire lesion, an even smaller difference was noted between colposcopy and biopsy. This finding suggests that biopsy can be used as a treatment in small lesions, especially in younger woman.

When there is more time between procedures, conization can find cases of high-grade lesions or cancer because the lesions progress during this waiting period. Higher-grade lesions or cancer can also be found by conization when the biopsy is performed incorrectly. Because there are several factors associated with cervical cancer, there is no exact answer regarding whether the time between procedures can affect the outcome. 18 Cervical lesion severity can be underestimated by biopsy, even if it is performed in the area of greatest suspicion; however, the large area analyzed by conization facilitates the correct diagnosis. 12 Furthermore, there is a lower possibility of cervical dysplasia progression because its evolution occurs slowly. 19

Although it was not possible to perform a statistical analysis, there was increased accuracy, sensitivity, specificity, and positive predictive value when more quadrants were involved in colposcopy. The increased accuracy and diagnostic agreement with more quadrants led to more correct diagnoses of high-grade lesions and cancer. Regarding microinvasive and invasive carcinoma, 82.35% of patients had lesion involvement in two or more quadrants. It was previously reported that the number of quadrants involved by lesions is related to the increased severity found by colposcopy and conization and faster progression to invasive cancer. 20 21

The possibility of false-negative results was greater when lesions involved only one quadrant, indicating that the lesion size might be related to better assessments and correct diagnoses. Songveeratham et al. 22 found an ∼ 15% discrepancy rate (underdiagnosis) between Pap test and conization results for high-grade lesions; this was related to the small size of high-grade lesions and presence of low-grade lesions.

For patients with microinvasive or invasive carcinoma found by conization, 94.1% had undergone colposcopy indicating high-grade findings or suspected invasion, and 88.2% had undergone biopsy indicating high-grade findings (negative results were found for two patients). With a small number of patients, there seems to be no difference between the findings of colposcopy and biopsy; therefore, biopsy was not important to exclude the diagnosis of microinvasive or invasive carcinoma, the literature shows that the sensitivity of colposcopy in the diagnosis of microinvasive carcinoma of the cervix was low. 23 Conversely, Baldauf et al. 18 found invasive lesions during conization of patients with high-grade lesions found by biopsy; therefore, they recommend conization for patients with high-grade lesions found by biopsy, regardless of colposcopy findings.

The cut-off age was because from that age, a hormonal drop (perimenopause) starts, and a greater atrophy of the epithelium of the vagina and cervix is observed. When women were divided into the younger than 40 years and 40 years or older groups, both diagnostic tests had decreased accuracy and increased overtreatment rates for high-grade lesions or cancer in older patients. Data have shown that the accuracy of colposcopy-directed biopsy is directly related to age; it is higher for patients younger than 30 years and progressively decreases with increasing age. 9 This is because older patients are often menopausal, have a greater degree of atrophy, and more often have type-3 transformation zones, where the endocervical component is not fully visible.

The main limitation of the study is its retrospective nature; therefore, we cannot exclude potential selection bias in the inclusion of cases. This also may have limited the availability of some important information. The sample size was small with only one quadrant involved by lesions and patients 40 years or older. Finally, the long period between colposcopy, biopsy, and conization may have impacted the natural course of the disease. Future studies should include a larger number of patients 40 years or older and those with one quadrant involved by lesions to confirm the results found in our study.

## Conclusion

Our results suggest that there is no difference between the accuracy of colposcopy and nor biopsy in diagnosing cervical intraepithelial lesions in the final result of conization. Patients who had the greatest benefit when biopsy was eliminated were those with high-grade lesions found by colposcopy, those with lesion involvement of two or more quadrants, and those younger than 40 years. Resource-saving strategies that lead to greater patient compliance without causing harm should be increasingly studied and implemented, especially in underdeveloped countries.
